# Retraction Note: CD276 enhances sunitinib resistance in clear cell renal cell carcinoma by promoting DNA damage repair and activation of FAK-MAPK signaling pathway

**DOI:** 10.1186/s12885-025-15511-z

**Published:** 2026-01-12

**Authors:** Zhi-yu Zhang, Jian-hao Xu, Jiang-lei Zhang, Yu-xin Lin, Jun Ou-Yang

**Affiliations:** 1 Department of Urology, The First Affiliated Hospital of Soochow University, Suzhou, Jiangsu 215000 China; 2 Department of Pathology, The First People’s Hospital of Kunshan, Suzhou, Jiangsu 215300 China

**Retraction Note: BMC Cancer 24, 650 (2024)**

**https://doi.org/10.1186/s12885-024-12402-7**

The Editors have retracted this article.

After publication, the corresponding author requested a correction to a duplicated image in Fig. 3c. However, further checks of the supplementary materials by the publisher found discrepancies between the full western blot scans presented and the expected molecular weights of the analyzed proteins. The authors were unable to provide a sufficient explanation and original raw data.

The editors therefore no longer have confidence in the data presented in the article.

All authors agree to the retraction.

